# Impact of *Lactobacillus johnsonii* CNCM I-4884 on canine giardiasis: a probiotic-based approach

**DOI:** 10.1186/s13071-025-07166-3

**Published:** 2025-12-06

**Authors:** Bruno Polack, Myriam Thomas, Alejandra Wu-Chuang, Lianet Abuin-Denis, Alejandro Cabezas-Cruz, Elsa Jacouton, Mohamed Mammeri, Isabelle Florent, Luis G. Bermúdez-Humarán

**Affiliations:** 1 Laboratoire de Santé Animale, Anses, INRAE, Ecole Nationale Vétérinaire d’Alfort, UMR BIPAR, 94700 Maisons-Alfort, France; 2 Micalis Institute, Domain de Vilvert, Université Paris-Saclay, INRAE, AgroParisTech, 78350 Jouy-en-Josas, France; 3 UMR 7245, Muséum National d’Histoire Naturelle, Centre National de La Recherche Scientifique, Sorbonne Universités, 75005 Paris, France

**Keywords:** *Lactobacillus johnsonii*, Probiotics, *Giardia intestinalis*, Giardiasis, Dogs

## Abstract

This study reports a new therapeutic approach for canine giardiasis, the most common intestinal protozoan infection caused by *Giardia intestinalis*. It is based on the use of the probiotic strain *Lactobacillus johnsonii* CNCM I-4884 and, in particular, its bile salt hydrolase enzymatic activities. Clinical trials in dogs demonstrated that daily administration of *L. johnsonii* CNCM I-4884 significantly reduced *Giardia* cyst shedding after 14 days. These results highlight the potential of this probiotic as a promising alternative to antimicrobials, such as nitroimidazoles or benzimidazoles, for the treatment of giardiasis in dogs. Moreover, they provide a novel approach for the veterinary industry to develop innovative products targeting this parasite. In addition to its direct anti-*Giardia* effect, *L. johnsonii* CNCM I-4884 may also act as an adjuvant therapy, supporting intestinal homeostasis, enhancing host defense mechanisms, and promoting recovery of gut balance during or after antiparasitic treatments. This dual role suggests that the strain could be considered not only as a complementary therapy but, in specific cases, as a potential stand-alone probiotic treatment for canine giardiasis.

**Graphical Abstract:**

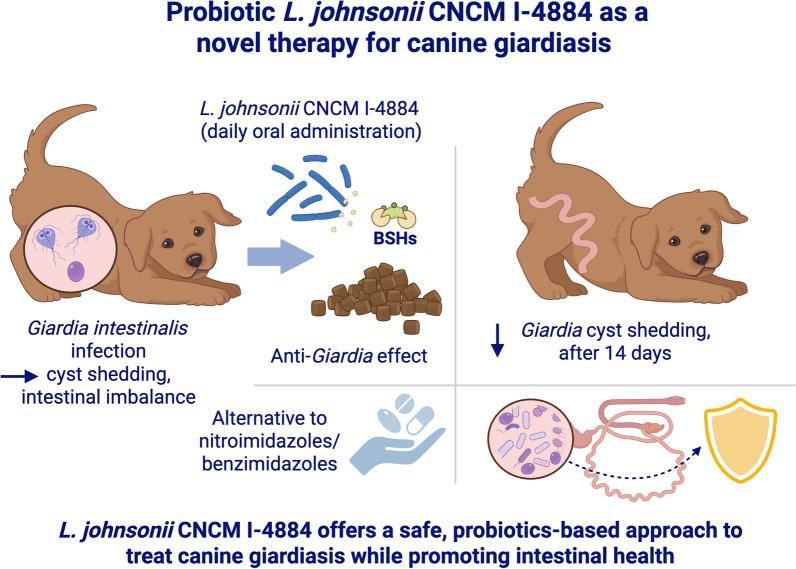

**Supplementary Information:**

The online version contains supplementary material available at 10.1186/s13071-025-07166-3.

*Giardia intestinalis* is a protozoan parasite that causes giardiasis, a common intestinal infection affecting humans and mammals worldwide [1]. Symptoms of giardiasis include diarrhea, abdominal pain, weight loss, and malabsorption, although infections are frequently asymptomatic [2]. *G. intestinalis* is classified into eight genetic groups, known as assemblages, with the following host preference: A and B (humans and other mammals, zoonotic), C and D (canids), E (ungulates), F (felines), G (rodents), and H (pinnipeds) [3]. However, these assemblages are not strictly host specific; canine genotypes have been observed in humans as well as in ruminants and pigs, and assemblages A, B, and F have also been found in dogs [3, 4].

Two recent meta-analysis studies estimated that the prevalence of giardiasis in dogs was, respectively, 13% [5] and 15.2% [6]. Nevertheless, this prevalence was nearly three times higher before 6 months of age than after and twice as high in strays/kennel dogs as in pet dogs [7]. The main treatments for *G. intestinalis* infections are benzimidazoles and nitroimidazoles. However, treatment failures are increasingly common, affecting 5-50% of cases, partly owing to the emergence of drug-resistant strains over the last 15 years [5, 6, 8, 9]. This growing resistance highlights the need for alternative therapeutic strategies.

Probiotics have emerged as a promising alternative for the prevention and treatment of giardiasis [10]. Our recent studies have demonstrated that the probiotic strain *Lactobacillus johnsonii* CNCM I-4884 can inhibit the growth of *G. intestinalis* both in vitro and in vivo using a murine model of giardiasis [11]. This protective effect is mostly mediated by bile salt hydrolase (BSH) enzymes, which convert conjugated bile acids into unconjugated forms that are toxic to the parasite [12, 13]. On the basis of this discovery, we patented a new therapeutic approach to fight giardiasis.

In this study, we validated the potential of this probiotic strain for veterinary use through a clinical trial in young Beagle dogs naturally infected with *G. intestinalis*. The animals were randomly selected, and initial infection was confirmed by centrifugal flotation with zinc sulfate and a positive IDEXX SNAP^®^ Giardia Test (SNAP-*Giardia* SNAP^®^ Giardia™, Idexx, France) 3 days before the experiment (D-3). Only healthy dogs excreting *Giardia* were included; prior treatments involved fenbendazole 2 weeks before D-3 and toltrazuril 3–4 days after weaning, with coproscopy confirming absence of other parasites. Dogs remained asymptomatic throughout the study to avoid ethical issues associated with treating sick animals, and no clinical signs requiring intervention occurred.

Before the trial, we optimized laboratory-scale production and storage conditions for the probiotic strain. Comparing freeze-dried and frozen preparations with glycerol, we found glycerol freezing to best preserve bacterial viability. The probiotic was then administered orally for 14 days at 1 × 10^10^ colony forming units (CFU)/day to 20 naturally infected puppies (mean age 7.2 weeks; 20 males, 20 females). A placebo group (*n* = 20) received glycerol alone under identical conditions. Fecal samples were collected individually at days 0, 4, 7, 11, and 14 post treatment, either refrigerated at 4 °C for cyst counting or frozen at −80 °C for quantitative polymerase chain reaction (qPCR) and microbiota analysis. *Giardia* cysts were quantified using direct immunofluorescence assays (DIF) with MeriFluor^®^ *Cryptosporidium/Giardia* (Meridian Bioscience, Italy), the international gold standard for cyst and trophozoite detection (see Fig. 1 for more detail). The study design allowed assessment of the probiotic anti-*Giardia* effect while maintaining ethical standards and scientific validity.

**Fig. 1:**
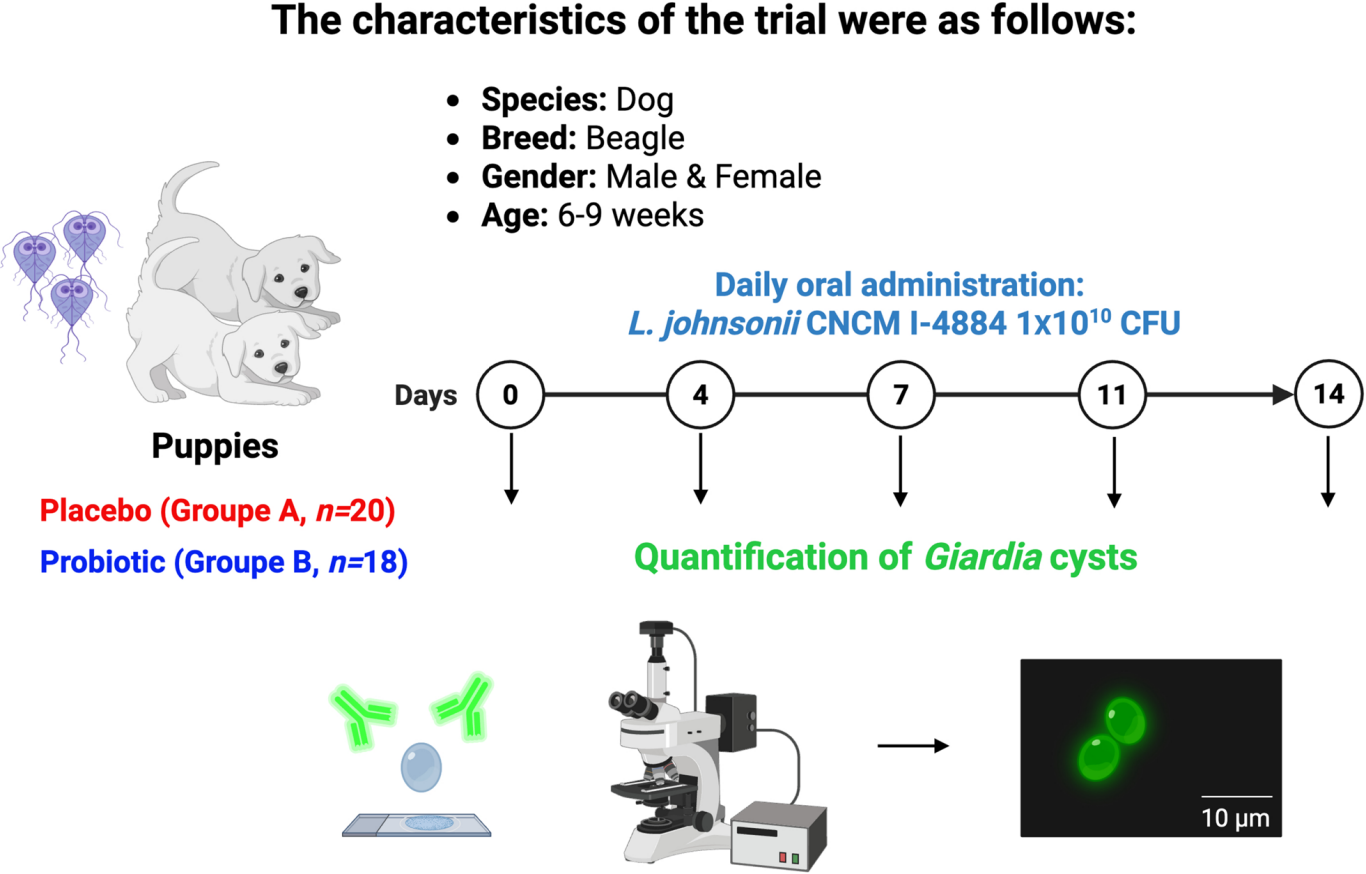
Characteristics of the clinical trial and experimental strategy. Created in BioRender. Bermudez, L. (2025) https://BioRender.com/614o83o

Briefly, 1 g aliquots of fresh fecal samples were diluted in 10 mL distilled water and filtered through three layers of surgical gauze. DIF assays were then performed in duplicate using 20 μL of this solution with Merifluor^®^ *Cryptosporidium*/*Giardia* kit (Meridian Bioscience, Italy). Whole slides were examined under a fluorescent microscope with 20× objective. As shown in Fig. 2A, *G. intestinalis* cyst counts showed a significant reduction in cyst numbers in the probiotic-treated group at days 7, 11, and 14 compared with the control group. In addition, the level of bacterial species *L. johnsonii* was determined by qPCR with the primers: Rw_ AGCATCTGTTTCCAGGTGTTATCC; Fw_ AGTCGAGCGAGCTAGCCTAGATG as previously described [14]. To quantify and normalize the data, we used the delta cycle threshold (CT) method with CT values obtained with the following primers: Rw_ CGCCACTGGTGTTCYTCCATATA; Fw_ AGCAGTAGGGAATCTTCCA [14] as reference gene. The results were normalized according to the weight of the fecal extract used for DNA extraction. Statistical analysis was performed using GraphPad Prism software (version 5) and a nonparametric Mann–Whitney statistical test. As shown in Fig. 2B, *L. johnsonii* species were detected in both groups (placebo and probiotic) at all sampling times. However, at day 0, no significant differences were observed between the two groups. Nonetheless, at the end of the study (day 14), the probiotic-treated group (B) exhibited a significant increase in the abundance of this species compared with the control group (A) (*P* = 0.0059). This increase aligns with the daily intake of the strain in the probiotic group.

**Fig. 2:**
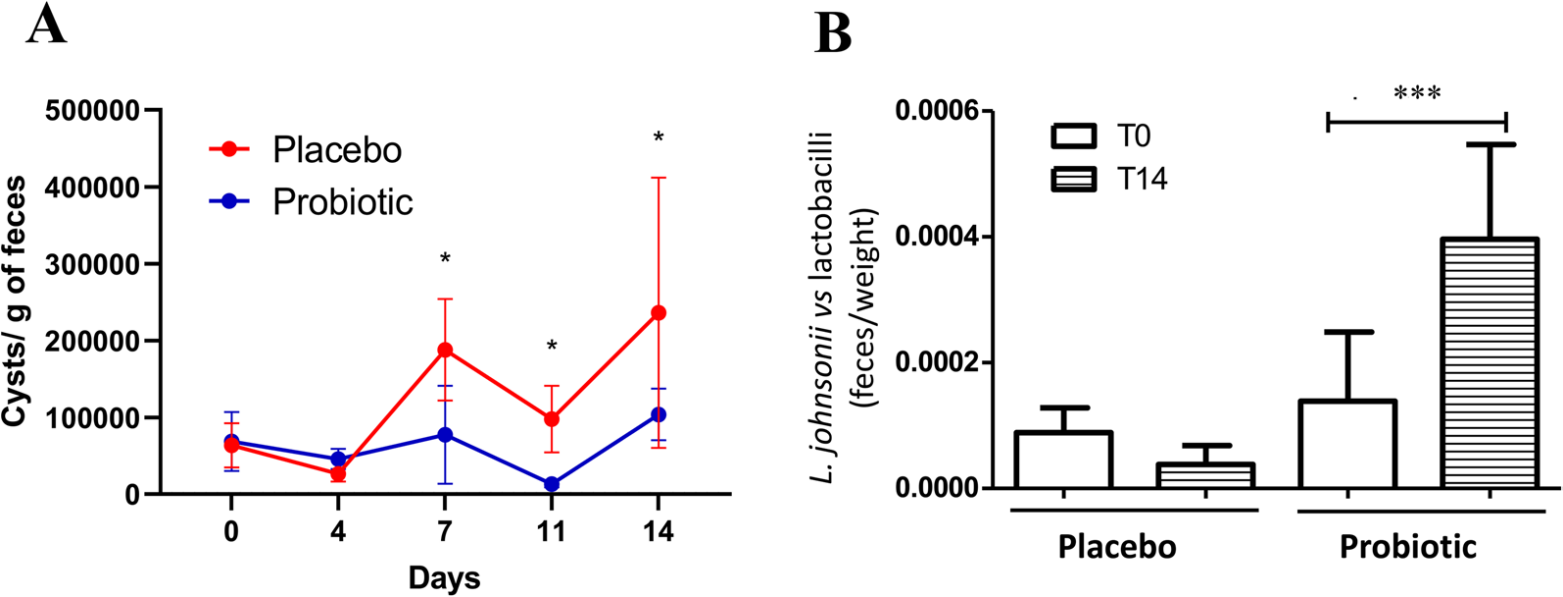
**A** Enumeration of *G. intestinalis* cysts in fecal samples after probiotic treatment. Values are mean ± standard error of the mean (SEM). **B** Levels of *L. johnsonii* in the feces of puppies treated with probiotics or placebo (^*^P < 0.05, ^***^P < 0.001)

In addition, fecal samples at two different time points (D0 and D14) were sequenced to determine the impact of our probiotic administration on gut microbiota composition. Raw sequences were de-interlaced, de-multiplexed, and processed in QIIME2 [15] using DADA2 [16] for de-noising and merging of reads. Amplicon sequence variants were aligned with MAFFT [17], and a phylogenetic tree was constructed with FastTree [18]. Taxonomic classification was performed using a naïve Bayes classifier trained on the SILVA 138 database [19]. Alpha and beta diversity indexes were calculated with the q2-diversity plugin in the QIIME2 environment. Alpha diversity analysis showed that both Shannon entropy (Fig. 3A) and Faith’s phylogenetic diversity (Fig. 3B) did not differ significantly between the probiotic-treated and placebo control groups. In contrast, beta diversity analysis revealed that probiotic treatment led to changes in the bacterial community composition and abundance, compared with the other three experimental groups, as measured by the Jaccard index (Fig. 3C, PERMANOVA, *q* < 0.05) and Bray–Curtis dissimilarity (Fig. 3D, PERMANOVA, *q* < 0.05). Taxa abundance differences were evaluated using the ALDEx2 package in R. Only the abundance of the taxon *Escherichia–Shigella* was significantly different among the different groups (Fig. 3E). Specifically, the abundance of *Escherichia–Shigella* was significantly higher in the probiotic-treated group at day 14 compared with the same group at day 0 (Fig. 3E, Kruskal–Wallis,* q* < 0.05). However, the abundance of *Escherichia–Shigella* at day 14 was not significantly different from the placebo group at the same day (Fig. 3E, Kruskal–Wallis,* q* > 0.05).

**Fig. 3:**
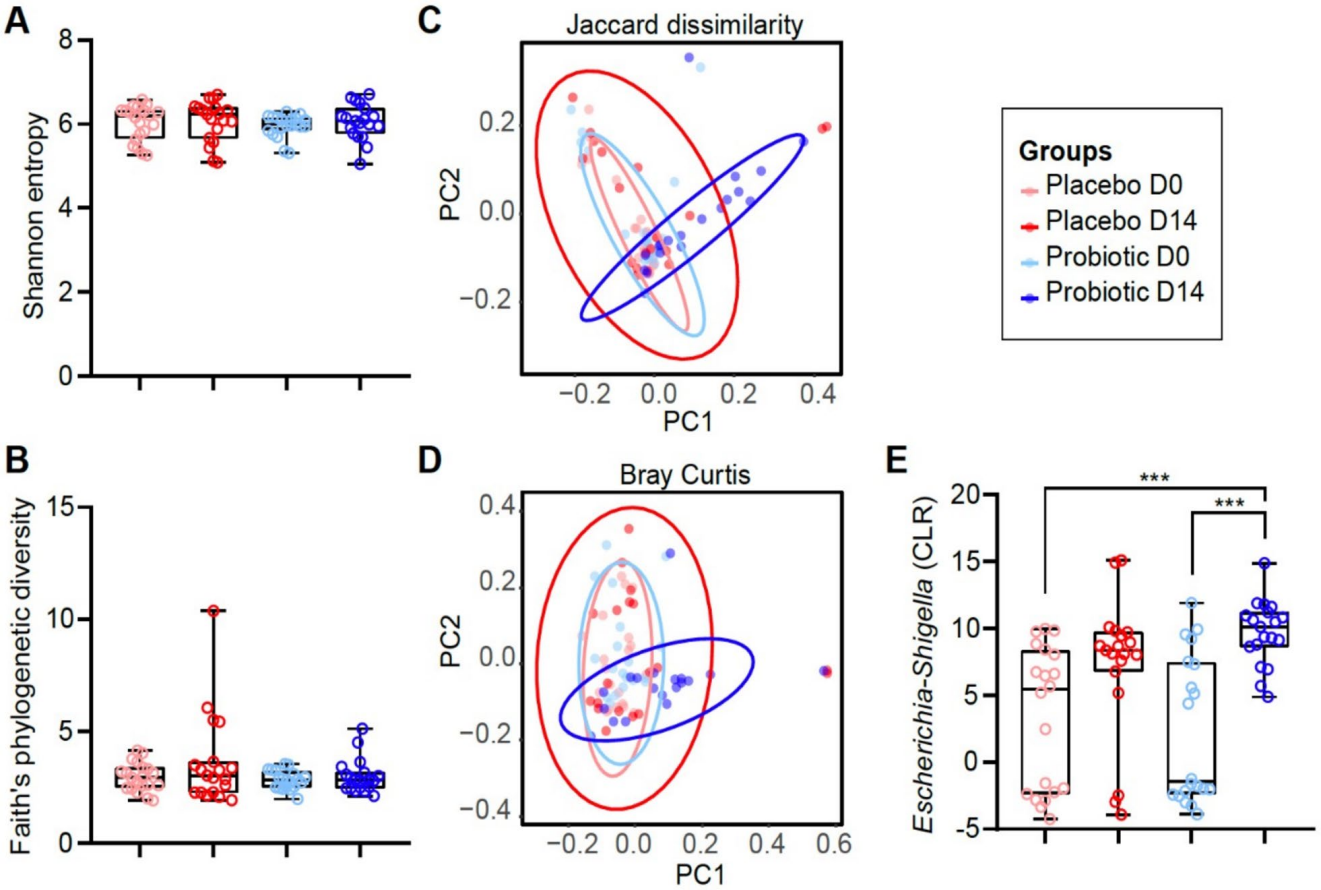
Probiotic impact on gut microbiota. Alpha diversity of the microbiota was measured using **A** Shannon entropy and **B** Faith phylogenetic indexes in dogs treated with probiotic compared with the placebo group. Beta diversity of dog microbiota was analyzed with **C** Jaccard, and **D** Bray–Curtis dissimilarity indexes to measure the similarity between the bacterial communities in probiotic compared with the placebo control group. **E** CLR values of the taxon *Escherichia–Shigella* whose abundance changed significantly between different treatment groups

In conclusion, our findings demonstrate that the probiotic strain *L. johnsonii* CNCM I-4884 is effective in controlling giardiasis in naturally infected dogs. This probiotic-based approach could serve as a valuable complement to existing registered drugs such as benzimidazole and metronidazole in the management of this widespread parasitic disease. In addition, gut microbiota analysis revealed that the efficacy of the probiotic is accompanied by changes in gut microbiota composition, including an increase in the *Escherichia*–*Shigella* taxon in treated dogs. An increase in *Escherichia* within the gut microbiota may be beneficial to the host, particularly when the strain is nonpathogenic, as it could help maintain a balanced microbial ecosystem or participate in competitive exclusion. For example, the probiotic *Escherichia coli* Nissle 1917 has been shown to reduce intestinal colonization by *Salmonella enterica Typhimiurium* by competing for iron [20]. Whether *Escherichia* plays a role in the observed reduction of *Giardia* in this study remains to be investigated. It is noteworthy, however, that the increase of *Escherichia–Shigella* abundance at day 14 was observed in both the probiotic-treated and placebo groups, suggesting that this change in abundance could be due to factors related to age or environment and not necessarily to the probiotic treatment.

Beyond its direct anti-*Giardia* activity, the use of *L. johnsonii* CNCM I-4884 may also provide secondary benefits to the host by modulating gut microbiota composition, enhancing mucosal defense mechanisms, and supporting recovery of gut homeostasis during or after antiparasitic therapy. Such adjuvant effects underline its potential as a complementary intervention alongside standard treatments, and in certain cases, as a promising stand-alone therapeutic option.

## Supplementary Information

Supplementary Material 1.

## Data Availability

Data supporting the main conclusions of this study are included in the manuscript.

## References

[1] Cernikova L, Faso C, Hehl AB. Five facts about *Giardia lamblia*. PLoS Pathog. 2018;14:e1007250. 10.1371/journal.ppat.1007250. 30261050 10.1371/journal.ppat.1007250 PMC6160191

[2] Adam RD. *Giardia duodenalis*: biology and pathogenesis. Clin Microbiol Rev. 2021;34:e0002419. 10.1128/CMR.00024-19. 34378955 10.1128/CMR.00024-19 PMC8404698

[3] Caccio SM, Lalle M, Svard SG. Host specificity in the *Giardia duodenalis* species complex. Infect Genet Evol. 2018;66:335–45. 10.1016/j.meegid.2017.12.001. 29225147 10.1016/j.meegid.2017.12.001

[4] Silva A, Martins FDC, Ladeia WA, Kakimori MTA, Lucas JI, Sasse JP, et al. First report of Giardia duodenalis assemblage F in humans and dogs in southern Brazil. Comp Immunol Microbiol Infect Dis. 2022;89:101878. 10.1016/j.cimid.2022.101878. 36108583 10.1016/j.cimid.2022.101878

[5] Arguello-Garcia R, Leitsch D, Skinner-Adams T, Ortega-Pierres MG. Drug resistance in *Giardia*: mechanisms and alternative treatments for giardiasis. Adv Parasitol. 2020;107:201–82. 10.1016/bs.apar.2019.11.003. 32122530 10.1016/bs.apar.2019.11.003

[6] Lalle M, Hanevik K. Treatment-refractory giardiasis: challenges and solutions. Infect Drug Resist. 2018;11:1921–33. 10.2147/IDR.S141468. 30498364 10.2147/IDR.S141468 PMC6207226

[7] Bouzid M, Halai K, Jeffreys D, Hunter PR. The prevalence of *Giardia* infection in dogs and cats, a systematic review and meta-analysis of prevalence studies from stool samples. Vet Parasitol. 2015;207:181–202. 10.1016/j.vetpar.2014.12.011. 25583357 10.1016/j.vetpar.2014.12.011

[8] Morch K, Hanevik K. Giardiasis treatment: an update with a focus on refractory disease. Curr Opin Infect Dis. 2020;33:355–64. 10.1097/QCO.0000000000000668. 32773501 10.1097/QCO.0000000000000668

[9] Kaufmann H, Zenner L, Benabed S, Poirel MT, Bourgoin G. Lack of efficacy of fenbendazole against *Giardia duodenalis* in a naturally infected population of dogs in France. Parasite. 2022;29:49. 10.1051/parasite/2022048. 36315102 10.1051/parasite/2022048 PMC9621113

[10] Travers MA, Florent I, Kohl L, Grellier P. Probiotics for the control of parasites: an overview. J Parasitol Res. 2011;2011:610769. 10.1155/2011/610769. 21966589 10.1155/2011/610769 PMC3182331

[11] Allain T, Chaouch S, Thomas M, Travers MA, Valle I, Langella P, et al. Bile salt hydrolase activities: a novel target to screen anti-*Giardia lactobacilli*? Front Microbiol. 2018;9:89. 10.3389/fmicb.2018.00089. 29472903 10.3389/fmicb.2018.00089 PMC5809405

[12] Travers MA, Sow C, Zirah S, Deregnaucourt C, Chaouch S, Queiroz RM, et al. Deconjugated bile salts produced by extracellular bile-salt hydrolase-like activities from the probiotic *Lactobacillus johnsonii* La1 inhibit *Giardia duodenalis* in vitro growth. Front Microbiol. 2016;7:1453. 10.3389/fmicb.2016.01453. 27729900 10.3389/fmicb.2016.01453 PMC5037171

[13] Allain T, Chaouch S, Thomas M, Vallee I, Buret AG, Langella P, et al. Bile-salt-hydrolases from the probiotic strain *Lactobacillus johnsonii* La1 mediate anti-giardial activity in vitro and in vivo. Front Microbiol. 2017;8:2707. 10.3389/fmicb.2017.02707. 29472895 10.3389/fmicb.2017.02707 PMC5810305

[14] Mayeur C, Gratadoux JJ, Bridonneau C, Chegdani F, Larroque B, Kapel N, et al. Faecal D/L lactate ratio is a metabolic signature of microbiota imbalance in patients with short bowel syndrome. PLoS ONE. 2013;8:e54335. 10.1371/journal.pone.0054335. 23372709 10.1371/journal.pone.0054335 PMC3553129

[15] Bolyen E, Rideout JR, Dillon MR, Bokulich NA, Abnet CC, Al-Ghalith GA, et al. Reproducible, interactive, scalable and extensible microbiome data science using QIIME 2. Nat Biotechnol. 2019;37:852–7. 10.1038/s41587-019-0209-9. 31341288 10.1038/s41587-019-0209-9 PMC7015180

[16] Callahan BJ, McMurdie PJ, Rosen MJ, Han AW, Johnson AJ, Holmes SP. DADA2: high-resolution sample inference from Illumina amplicon data. Nat Methods. 2016;13:581–3. 10.1038/nmeth.3869. 27214047 10.1038/nmeth.3869 PMC4927377

[17] Katoh K, Misawa K, Kuma K, Miyata T. MAFFT: a novel method for rapid multiple sequence alignment based on fast Fourier transform. Nucleic Acids Res. 2002;30:3059–66. 10.1093/nar/gkf436. 12136088 10.1093/nar/gkf436 PMC135756

[18] Price MN, Dehal PS, Arkin AP. FastTree 2--Approximately maximum-likelihood trees for large alignments. PLoS ONE. 2010;5:e9490. 10.1371/journal.pone.0009490. 20224823 10.1371/journal.pone.0009490 PMC2835736

[19] Yarza P, Yilmaz P, Pruesse E, Glockner FO, Ludwig W, Schleifer KH, et al. Uniting the classification of cultured and uncultured bacteria and archaea using 16S rRNA gene sequences. Nat Rev Microbiol. 2014;12:635–45. 10.1038/nrmicro3330. 25118885 10.1038/nrmicro3330

[20] Deriu E, Liu JZ, Pezeshki M, Edwards RA, Ochoa RJ, Contreras H, et al. Probiotic bacteria reduce *Salmonella typhimurium* intestinal colonization by competing for iron. Cell Host Microbe. 2013;14:26–37. 10.1016/j.chom.2013.06.007. 23870311 10.1016/j.chom.2013.06.007 PMC3752295

